# Development of a highly purified tick-borne encephalitis vaccine: A personal historical account

**DOI:** 10.1007/s00508-023-02240-1

**Published:** 2023-06-30

**Authors:** Franz X. Heinz

**Affiliations:** Center for Virology, Medical University of Vienna, Kinderspitalgasse 15, 1090 Vienna, Austria

**Keywords:** Second generation vaccine, History of vaccine development, Continuous-flow zonal ultracentrifugation, High vaccine purity, Low vaccine reactogenicity

## Abstract

Before the advent of a vaccine, infections with tick-borne encephalitis (TBE) virus in Austria led to the hospitalization of several hundred and, due to underreporting, possibly more than thousand patients with severe neurological disease every year. In the late 1960s and early 1970s, this country had the highest recorded morbidity of TBE in Europe, but similar endemic risk areas exist in many other European countries as well as Central and Eastern Asia. In this article, I describe my personal recollections of the development of a highly purified TBE vaccine in the late 1970s, to which I contributed as a young post-doctoral scientist mentored by Christian Kunz (then director of the Institute of Virology at the Medical Faculty, University of Vienna) in a collaboration with the Austrian biopharmaceutical company Immuno. Low reactogenicity of the newly developed vaccine was a prerequisite for mass vaccination campaigns in Austria that started in the early 1980s. Because of its excellent immunogenicity, broad application of the highly purified vaccine paved the way for a dramatic reduction of the incidence of TBE in Austria, which is outstanding in Europe and referred to as an Austrian success story of immunoprophylaxis.

## Introduction

Tick-borne encephalitis (TBE) is the most important arthropod-transmitted viral disease of humans in Europe, with its geographical distribution extending to large parts of Central and Eastern Asia [1]. The causative agent (TBE virus) is maintained in its natural animal reservoir by circulation between rodents and ticks. Humans are infected incidentally only, usually by the bites of virus-infected ticks or occasionally through the consumption of infected raw milk or milk products from goat, sheep or cows [1]. In some of the most affected countries, TBE represents a serious public health problem. According to the World Health Organization (WHO) 10,000–12,000 cases are officially recorded in Europe and Asia [2] but considerable underreporting may be assumed.

Austria holds an exceptional position concerning the immunoprophylaxis of this disease. Vaccination coverage has reached more than 80% of the total population and more than 90% in certain high-risk areas [3], by far exceeding that of other European countries with similar incidences of TBE [4–6]. The high degree of protection conferred by the vaccine led to a dramatic decline of annual cases, an achievement referred to as the Austrian experience of TBE vaccination [7]. Success was made possible by annual campaigns for raising TBE awareness and mass vaccination with a highly purified vaccine developed in the late 1970s that was well tolerated and protective [3, 7]. This was a second-generation vaccine following a vaccine developed between 1971 and 1973, which appeared to be sufficiently immunogenic but caused considerable side reactions that were an obstacle to mass vaccination and its use in children, necessitating new developments.

In 1973, I started my PhD thesis at the Institute of Virology, Medical Faculty, University of Vienna, under the supervision of its director Christian Kunz, and became directly involved in the development of a second-generation TBE vaccine in a collaboration between Christian Kunz and the Austrian biopharmaceutical company Immuno. In this article, I present my personal account of this crucial stage of generating a substantially improved TBE vaccine that was suitable for application on a large scale and represented a cornerstone of the success achieved with TBE vaccination in Austria.

## The first-generation vaccine

A first phase of vaccine development took place between 1971 and 1973, in a collaboration between Christian Kunz and the Microbiological Research Establishment (MRE) in Porton Down, England. Since I joined Christian Kunz for my PhD thesis only in 1973, my knowledge of the sequence of events leading to the resulting experimental TBE vaccine is indirect and largely based on oral communication and documentation in the literature [7, 8]; however, I already witnessed directly the arrival of first batches of vaccine at our institute in Vienna in spring 1973 and the following start of small-scale immunization trials.

In 1971, Christian Kunz had become the first full professor of virology in Austria and director of the newly founded Institute of Virology at the Medical Faculty, University of Vienna. Through his research on the epidemiology and diagnostics of TBE, he realized the extent of the public health problem imposed by this disease and had the ambition of making a vaccine available, especially for people with an occupational risk of contracting TBE, e.g., farmers and forestry workers [7]. In the 1960s, he had stayed for 2 years at the Rockefeller Laboratories in New York and developed an excellent reputation as a scientist in the area of what we would call emerging viruses today. Making use of his international network, he contacted Gordon Smith, in 1971 director of the MRE in Porton, knowing that this institution had developed an experimental vaccine against Louping Ill virus. This virus is a close relative of TBE virus that occurs in Great Britain and primarily affects sheep but has marginal importance as a human disease agent [9].

In his discussions with the British stakeholders (which also involved representatives of The Wellcome Foundation Ltd. as a commercial company), Christian Kunz proposed the development of a TBE vaccine for Austria’s needs using the Louping Ill vaccine technology and an Austrian isolate of TBE virus as a starting point. His initiative fell on fertile ground and an experimental TBE vaccine was developed between 1971 and 1973, with James Keppie as the responsible scientist at MRE in close collaboration with Christian Kunz in Vienna [7]. Vaccine production at Porton consisted of growing the virus in suspensions of minced chick embryos, partial purification of infectious virus by hydroxyapatite chromatography and inactivation by formaldehyde. Aluminium hydroxide was added as an adjuvant. The first small batch of this vaccine arrived in Vienna in April 1973, just when I had started my PhD thesis in March. On 25 April Christian Kunz and his co-worker Hanns Hofmann vaccinated each other with the first two doses of vaccine [7], before the start of a small-scale clinical trial and the vaccination of 30,000 persons in 1974 and 1975, primarily people with an occupational risk of contracting TBE [10].

As the vaccine was still experimental in nature, a pharmaceutical company was needed to obtain market approval and to commercialize the product. Obviously, The Wellcome Foundation Ltd. would have been a logical candidate based on its involvement in LI and TBE vaccine development at MRE in Porton; however, very much to the disappointment of Christian Kunz, their responsible officers were not convinced of an appropriate market potential and decided to step out of the project in 1973, leaving him with the problem of looking for an alternative industrial partner. The problem was fortunately solved soon, at the end of 1973, when Christian Kunz could convince Otto Schwarz, together with Johann Eibl one of the directors and founders of the Austrian biopharmaceutical company Immuno, to step in and to take the risk of moving the experimental vaccine towards commercialization. Immuno contracted the MRE in Porton with the production of the bulk material for the vaccine, which was then processed and filled into ampoules at the production facility of Immuno in Vienna. Market approval in Austria was finally obtained by Immuno in 1976 [7].

The vaccine proved to be sufficiently immunogenic, as revealed by the demonstration of TBE virus-specific antibodies in most of the vaccinees [10], but a high rate of undesired local and systemic side reactions was a matter of concern [7, 8]. In addition to that, I remember many discussions with Christian Kunz on variations of potency among individual batches of vaccine, suggesting problems of consistency and standardization in the production process.

## Phase 2 of vaccine development—The highly purified vaccine

The frequency of side reactions observed with the first-generation vaccine was the main driving force behind further development. In this context, Immuno started a collaborative project with Christian Kunz and me to develop an improved product that would meet higher standards and comply with modern requirements.

Our scientific competence and potential for improving the vaccine was founded by the early initiative of Christian Kunz to invest in basic research on TBE virus. He personally sponsored my PhD thesis on the growth, purification and molecular characterization of the virus. Together we published our new data on the properties of TBE virus, and especially its envelope glycoprotein as the main protective antigen, in a series of publications [11–19]. Most importantly, in 1976 we were able to demonstrate that highly purified inactivated virus preparations did not exhibit any pyrogenicity in the established rabbit animal model, even at several-fold higher concentration of the viral antigen than present in the current vaccine. This important finding suggested that the fever reactions observed with the first-generation vaccine were not due to the immunogen itself (i.e., the inactivated virus) but likely caused by contaminants from the cell substrate and insufficient purity achieved during the production process. We therefore concluded that it should be possible to reduce reactogenicity substantially by developing a vaccine that contained the inactivated virus in a highly purified form.

### Inspiration from outside TBE virus research

In the mid-1970s, new influenza vaccines were considered a gold standard and represented the state of the art for guidance. We became familiar with the development of these vaccines and obtained deep insights into their design through our connections to researchers at the Sandoz Research Institute in Vienna. They had developed a next-generation influenza vaccine, based on the viral envelope proteins only [20], and Christian Kunz had conducted clinical trials on its immunogenicity and reactogenicity [21]. Throughout my PhD thesis, the personal interaction with the influenza scientists at the Sandoz Research Institute was inspiring and very helpful, fostering my understanding of modern virus research and making me familiar with a multitude of techniques that I could apply to my work on TBE virus.

One of these techniques was continuous-flow zonal ultracentrifugation, which was used for influenza virus purification in the course of vaccine development and production. This technology allows the simultaneous concentration and purification of viruses in a continuous process that is scalable for industrial production. In January 1977, I got the opportunity to carry out two experimental runs with the ultracentrifuge at the Sandoz Research Institute using inactivated TBE virus I had produced in our laboratories after growing the virus in primary chick embryo cells. Analyses of these experiments at the institute were encouraging, although many questions remained unresolved and needed detailed investigation in a specifically dedicated project.

### The second-generation TBE vaccine project

Several aspects of our work on TBE virus were crucial for exploring alternative procedures towards an improved TBE vaccine. These included the demonstration that the inactivated virus (constituting the immunogen of the vaccine) was not pyrogenic at concentrations needed (see above), as well as methods for detecting and quantifying the inactivated viral antigen. The latter aspect was especially important for designing new processes of vaccine production. In the first-generation vaccine produced at the MRE in Porton, partial purification was carried out with infectious virus suspensions, and standardization of the antigen concentration relied on infectivity measurements during the procedure; however, purification of a highly pathogenic infectious virus in a large-scale industrial process would require an enormous operating expense for biosafety containment and therefore should best be avoided.

Based on these considerations, Immuno ventured into a project for vaccine improvement, which included the evaluation of continuous-flow zonal ultracentrifugation for large-scale industrial purification of inactivated TBE virus. For that purpose, in 1977, they organized a mission for me and Johann Fauma (scientist at Immuno and in charge of TBE vaccine production) to the MRE in Porton, their contract partner for producing the bulk material of the vaccine then available. We obtained access to a continuous-flow zonal ultracentrifuge at the MRE and technical support, including the provision of the same starting material for our experimental ultracentrifugation runs that was also used for the conventional production, i.e., supernatants from infected chick embryo cells before purification. I was primarily in charge of defining the scientific questions to be asked and establishing the details of the work plan, and, in close coordination, Johann Fauma primarily took care of aspects relevant to industrial production and the Immuno agenda at Porton.

For the project, we conducted two series of experiments, the first in July and the second in November 1977. All samples collected from these experiments were sent to Vienna through Immuno for detailed analyses, using previously established methods for quantifying both the viral antigen and impurities throughout the purification process, as well as for measuring protective potency in a mouse animal model. The results (including recovery rates of viral antigen and immunogenicity) were highly convincing and Immuno swiftly invested in the new technology and implemented continuous-flow zonal ultracentrifugation for production at its industrial plant in Vienna. Already in 1979, the zonally purified vaccine replaced the old one on the Austrian market [22].

The newly developed method combined several innovative steps so that Immuno could file a patent application in 1978. The patent was granted in Austria in 1980, followed by several other countries, including Germany, Czechoslovakia, Great Britain, Switzerland, France, Italy, Slovenia, Sweden, and the Soviet Union. Details of the process were published by Heinz et al. in 1980 [23], demonstrating that the purity of the second-generation vaccine was more than 95-fold higher than that of the first-generation vaccine. Clinical trials yielded excellent data on immunogenicity combined with a substantial reduction of reactogenicity, as documented by the corresponding publication of Kunz et al. in 1980 [8].

For my published contributions in the development of a highly purified TBE vaccine as academic partner of Christian Kunz, I was awarded two national prizes in Austria: 1. The Merck Sharp&Dohme (MSD) Vaccine Prize 1983 for the design of immunoassays to quantify inactivated TBE virus [19]. 2. The HERBA Prize 1983 for the publication on the use of continuous-flow zonal ultracentrifugation for the preparation of a highly purified TBE vaccine [23].

More than 15 years after development of the new vaccine, in 1996, the international company Baxter took over Immuno, and in 2014, Baxter’s vaccine portfolio was acquired by Pfizer, which remained the producer of this TBE vaccine until today. During these years, changes were made to the industrial manufacturing process, including a switch from mouse brain-derived virus to chick embryo cell-derived virus for inoculating production cultures of primary chick embryo cells, and the removal of merthiolate as a preservative [24]. Continuous-flow zonal ultracentrifugation, however, represents the heart of the production and purification process until today and is integrated in a high-tech setting of modern state-of-the-art vaccine manufacturing at the Pfizer production plant in Orth/Donau near Vienna. A similar vaccine but using different stabilizers and based on a German TBE virus isolate, grown in primary chick embryo cells and also purified by continuous-flow zonal ultracentrifugation, was introduced in Germany by the Behringwerke AG in 1991 [24].

### Success of the highly purified vaccine on large-scale application in Austria

Evaluation of the effectiveness of TBE vaccination under real world conditions was enabled by the meticulous analysis of every single case of TBE in Austria and the documentation of its vaccination history [7]. This work was started by Christian Kunz and, after his retirement in 1995, continued by the institute he had founded until today. Patient data were combined with the figures on vaccination coverage in Austria, revealing a field effectiveness well above 90% [7]. The high rate of protection resulted in a continuous decline of TBE cases parallel to the increase of vaccination coverage. In contrast, the incidence of TBE in the unvaccinated population [25] as well as in neighboring countries with a comparably low vaccination rate remained unchanged [4, 25]. Referring to an Austrian success story [3] thus appears to be justified in this context.

## Conclusion

In summary, I was privileged even as a very young scientist, to take part in a fascinating journey from basic research to applied industrial development that led to a potent and well-tolerated vaccine, making immunization against tick-borne encephalitis (TBE) an outstanding achievement in Austria. This success was the result of a fruitful collaboration between academia and Immuno as a pioneer of Austrian biotechnological companies, founded in 1960 with its headquarters and production facilities in Vienna. Christian Kunz played a central role in vaccine development and is certainly the father of TBE vaccination in Austria. He initiated the development of a first experimental vaccine and realized early on that more fundamental science and knowledge about the virus itself would be necessary to aim at a modern vaccine that complied with the requirements of large-scale vaccination. I was very happy to join him in this endeavor and his collaboration with Immuno, a company ready to take entrepreneurial risk in bringing a first-generation vaccine to the market and in developing and producing a substantially improved next-generation vaccine.

Not least, my expertise of producing large amounts of highly purified TBE virus laid the foundation for one of the most important scientific projects in my research activities to follow. In a collaboration with Stephen Harrison and Felix Rey at Harvard University, we provided the envelope glycoprotein isolated from purified infectious TBE virus (produced at the Institute of Virology independent of continuous-flow zonal ultracentrifugation and vaccine production described above) for X‑ray structure determination, leading to the discovery of a previously unknown and completely new class of viral surface proteins [26]. This and following scientific projects on the structure, function, epidemiology and diagnostics of TBE and related viruses paved the way for my further career at the university, to become full professor of virology in 1999 and director of the Institute of Virology from 2004 (when the Medical Faculty of the University of Vienna became transformed into the Medical University of Vienna) until my retirement in 2015.
